# A Hyaluronan and Platelet-Rich Plasma Hydrogel for Mesenchymal Stem Cell Delivery in the Intervertebral Disc: An Organ Culture Study

**DOI:** 10.3390/ijms22062963

**Published:** 2021-03-15

**Authors:** Fabrizio Russo, Luca Ambrosio, Marianna Peroglio, Wei Guo, Sebastian Wangler, Jan Gewiess, Sibylle Grad, Mauro Alini, Rocco Papalia, Gianluca Vadalà, Vincenzo Denaro

**Affiliations:** 1 Laboratory for Regenerative Orthopaedics, Orthopaedic and Trauma Surgery Unit, Departmental Faculty of Medicine and Surgery, Campus Bio-Medico University of Rome, Via Alvaro del Portillo 200, 00128 Rome, Italy; fabrizio.russo@unicampus.it (F.R.); l.ambrosio@unicampus.it (L.A.); r.papalia@unicampus.it (R.P.); denaro@unicampus.it (V.D.); 2 AO Research Institute Davos, Clavadelerstrasse 8, 7270 Davos, Switzerland; marianna.peroglio@aofoundation.org (M.P.); guo0wei@hotmail.com (W.G.); sebastian-wangler@gmx.ch (S.W.); jan.gewiess@aofoundation.org (J.G.); sibylle.grad@aofoundation.org (S.G.); mauro.alini@aofoundation.org (M.A.); 3 Department of Orthopaedic Surgery and Traumatology, Inselspital, Bern University Hospital, University of Bern, 3012 Bern, Switzerland

**Keywords:** hyaluronic acid, platelet-rich plasma, mesenchymal stem cells, intervertebral disc, organ culture, ex vivo

## Abstract

The purpose of the present pilot study was to evaluate the effect of a hydrogel composed of hyaluronic acid (HA) and platelet-rich plasma (PRP) as a carrier for human mesenchymal stem cells (hMSCs) for intervertebral disc (IVD) regeneration using a disc organ culture model. HA was mixed with batroxobin (BTX) and PRP to form a hydrogel encapsulating 1 × 10^6^ or 2 × 10^6^ hMSCs. Bovine IVDs were nucleotomized and filled with hMSCs suspended in ~200 μL of the PRP/HA/BTX hydrogel. IVDs collected at day 0 and nucleotomized IVDs with no hMSCs and/or hydrogel alone were used as controls. hMSCs encapsulated in the hydrogel were also cultured in well plates to evaluate the effect of the IVD environment on hMSCs. After 1 week, tissue structure, scaffold integration, hMSC viability and gene expression of matrix and nucleus pulposus (NP) cell markers were assessed. Histological analysis showed a better preservation of the viability of the IVD tissue adjacent to the gel in the presence of hMSCs (~70%) compared to the hydrogel without hMSCs. Furthermore, disc morphology was maintained, and the hydrogel showed signs of integration with the surrounding tissues. At the gene expression level, the hydrogel loaded with hMSCs preserved the normal metabolism of the tissue. The IVD environment promoted hMSC differentiation towards a NP cell phenotype by increasing cytokeratin-19 (KRT19) gene expression. This study demonstrated that the hydrogel composed of HA/PRP/BTX represents a valid carrier for hMSCs being able to maintain a good cell viability while stimulating cell activity and NP marker expression.

## 1. Introduction

Low back pain (LBP) is mainly caused by intervertebral disc degeneration (IDD). Current treatment strategies include both conservative and surgical approaches. However, none of these can hamper or arrest IDD [1]. Thus, there is an ongoing effort to develop novel therapeutic tools which target the biological cascades involved in the degenerative process in order to prevent the invasiveness of spine surgery. Intradiscal stem cell application has been introduced as a promising regenerative approach, both in vitro and in vivo [2]. In particular, bone marrow mesenchymal stem cells (MSCs) hold the capacity to differentiate towards nucleus pulposus (NP)-like cells [3] and secrete a range of cytokines which induce an anabolic response in resident NP cells following intradiscal application [4]. However, there is an ongoing debate whether cells transplanted in cell suspensions survive the harsh environment found in degenerated IVDs [5]. It has been shown that encapsulation of MSCs within a hydrogel carrier can protect the cells and yield regenerative effects on degenerated IVDs in vivo [6]. However, several factors must be considered regarding the choice of the appropriate carrier and the selection of the most adequate transplantation method [7,8].

Platelet-rich plasma (PRP) is a concentrate of growth factors, plasma proteins and platelets (PLTs) obtained from the centrifugation of autologous whole blood. Due to its potential regenerative effects, it has emerged as a promising therapeutic tool in orthopaedics both in acute and chronic diseases [9]. PRP’s biological activity depends on the presence of several growth factors contained in α-granules stored inside PLTs, which are released upon PLT activation and rupture [10]. Moreover, PRP induces resident cells to synthesize additional biological factors and exerts an anti-inflammatory effect via blocking the nuclear factor kappa-light-chain-enhancer of activated B cells (NF-κB) pathway in different cell types [11]. In addition, PRP has been demonstrated to increase NP cell viability and matrix protein synthesis thus being able of slowing down IDD in animal models [12,13].

Hyaluronic acid (HA) is a long-chained nonsulfated glycosaminoglycan with viscoelastic properties naturally occurring in most extracellular matrices, especially in cartilage, providing tissues with resistance to compression, lubrication as well as promoting cell adherence, differentiation towards a chondrogenic phenotype and ECM protein synthesis [14]. For these reasons, HA has been widely utilized in the nonsurgical treatment of osteoarthritis (OA) to promote joint lubrication and cartilage repair, due to its rheological and anti-inflammatory features [15]. Furthermore, HA is one of the main components constituting the native NP and has therefore been used as a cell carrier for IVD regeneration in numerous pre-clinical studies [16], which demonstrated that HA could restore disc height and promote NP resident cell viability and matrix synthesis [17].

Due to the complementary beneficial effects of both components, the application of HA/PRP blends has been considered promising for the conservative treatment of OA.

We have previously shown that a hydrogel composed of HA and PRP blended with batroxobin (BTX) as a gelling agent is an efficacious MSC carrier for NP regeneration in vitro. The scaffold could promote MSC proliferation, viability and differentiation towards a NP-like phenotype while showing favorable rheological properties which encourage its clinical use [18].

In order to validate this hydrogel and promote its translation to an in vivo application, we used an ex vivo bovine organ culture model to test the behavior of the HA/PRP/BTX hydrogel within the IVD environment by assessing tissue structure, scaffold integration, MSC viability following transplantation and gene expression. As whole human IVDs are difficult to obtain, IVDs harvested from large animals have been widely employed due to their structural (size, cell populations, ECM composition) and functional analogies with the human counterpart [19]. Our group has formerly developed a bioreactor model to test hMSC transplantation into bovine IVDs for disc regeneration [20]. To date, IVD organ culture bioreactors have been shown to accurately reproduce the physiological disc microenvironment at a high degree of approximation, thus allowing to further explore IVD biology as well as to test innovative treatment approaches [21].

In the present pilot study, we aimed to evaluate the capacity of the previously validated HA/PRP/BTX hydrogel to support hMSC survival and IVD cell viability as well as to influence ECM anabolism and nucleopulpogenic differentiation of resident cells following intradiscal transplantation.

## 2. Results

### 2.1. hMSC and IVD Cell Viability and Distribution

**Figure 1:**
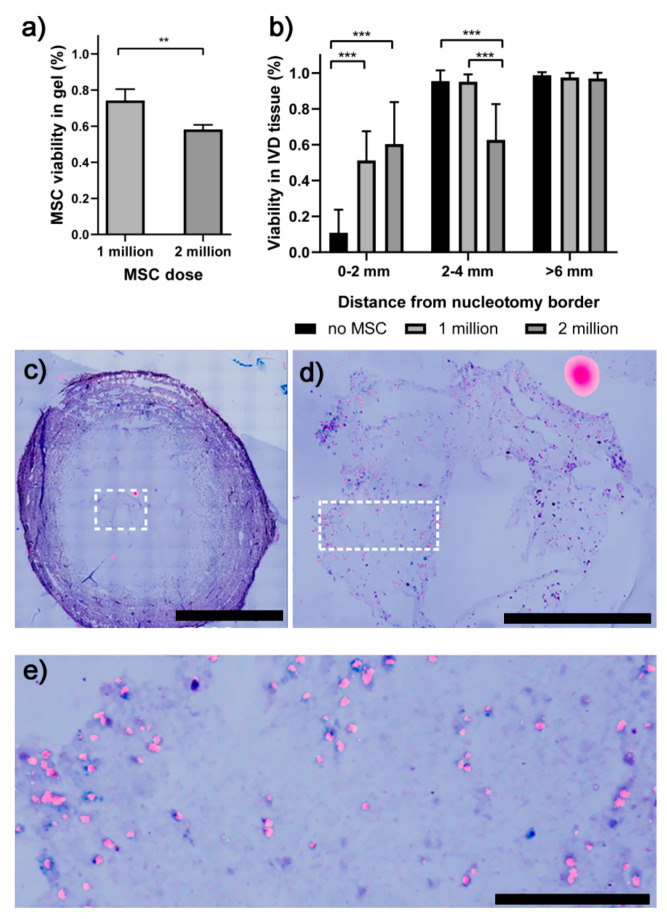
Viability of the hMSCs and the IVD cells following one week of culture assessed by lactate dehydrogenase (LDH)/ethidium homodimer 1. (**a**) hMSC viability inside the gel was inversely correlated with hMSC dose. (**b**) IVD cell viability was improved in the presence of hMSCs in the region adjacent to the gel. (**c**) A representative section of a nucleotomized IVD treated with the hydrogel containing 1 million hMSCs. (**d**) Magnified view of the hydrogel region (represented by the dotted rectangle in a). (**e**) Magnified view of the hydrogel region represented by a dotted rectangle in d. ** *p* < 0.01, *** *p* < 0.001. hMSCs = human mesenchymal stem cells; IVD = intervertebral disc; LDH = lactate dehydrogenase. Scale bars = 5000 μm (upper left side), 1000 μm (upper right side), 200 μm (bottom).

Cell viability was higher when 1 million of hMSCs were supplied into the disc using the HA/PRP carrier as compared to the 2 million hMSCs group (Figure 1a). This applied to both hMSC viability inside the gel and IVD cell viability in the surrounding tissue at 2–4 mm distance from the nucleotomy border (Figure 1a,b). Interestingly, the viability in the tissue adjacent to the nucleotomized and refilled region (0–2 mm distance) was similar with a dose of 1 or 2 million hMSCs, but was significantly different further away from the nucleotomy (2–4 mm distance). These trends were consistent for the experiments performed with two different bovine tails (the viability in the IVD tissue in the 2–4 mm region from the nucleotomy border was 2- to 3-fold higher when 1 million hMSCs versus 2 million hMSCs were supplied). The hydrogel only control group without hMSCs presented a markedly reduced cell viability adjacent to the nucleotomy area (Figure 1b). Representative images of the lactate dehydrogenase (LDH)/ethidium homodimer-stained sections of nucleotomized IVDs treated with MSC-loaded hydrogels are represented in Figure 1c–e.

**Figure 2:**
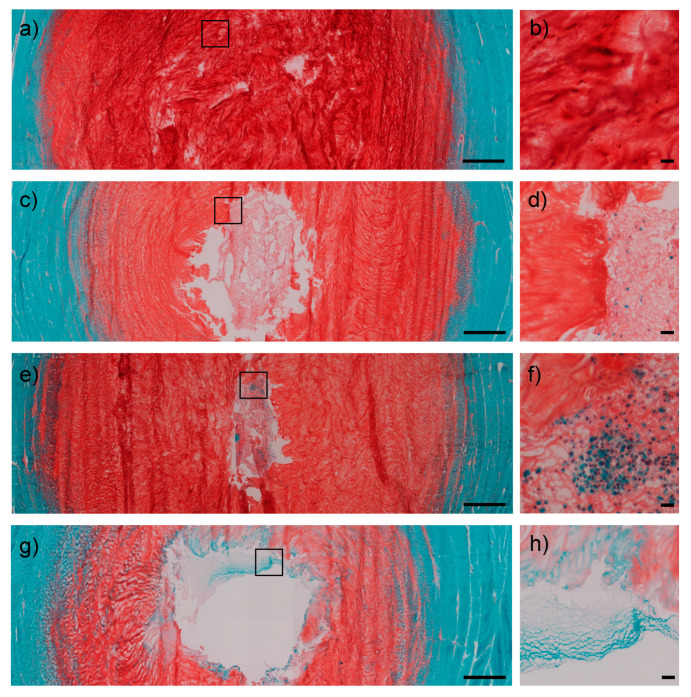
Safranin O/Fast Green stained transverse sections of IVDs. Control intact group showed structural integrity of the NP both at low (1.25×) and high (20×) magnifications (**a**,**b**). IVDs treated with 1 million hMSCs displayed a substantial retention of the scaffold within the NP defect (**c**), with a Safranin O staining intensity predictive of proteoglycan retention. Furthermore, the hydrogel appeared to be populated by cells and integrated with the surrounding tissues in highlighted regions (**d**). IVDs injected with 2 million hMSCs exhibited an even higher scaffold integration (**e**), with evidence of cell clusters and continuity with the remaining NP tissue (**f**). Otherwise, the control group (nucleotomy refilled with gel without MSCs) showed decreased cellularity around the defect (**g**,**h**). Scale bars = 1000 μm (overviews, left side); 50 μm (close-up views, right side). IVD = intervertebral disc; NP = nucleus pulposus; hMSCs = human mesenchymal stem cells.

After 1 week of culture, Safranin O/Fast Green staining confirmed scaffold retention within the NP cavity in all samples. The hydrogel showed signs of integration with the surrounding NP tissue in IVDs injected with both 1 and 2 million hMSCs (Figure 2c–f) and proteoglycan deposition, while this was not the case in the gel control group (Figure 2g,h).

### 2.2. The Effects of hMSCs within the HA/PRP/BTX Hydrogel on Disc Cell Phenotype

**Figure 3:**
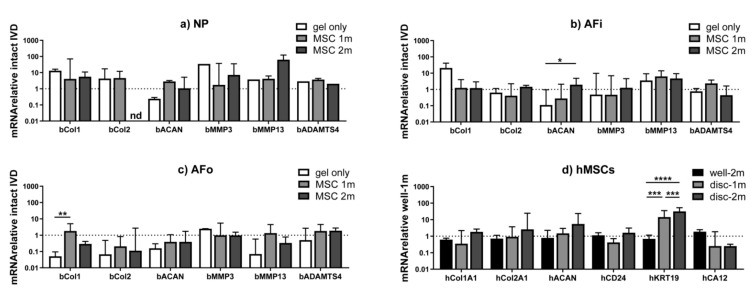
Anabolic, catabolic and NP differentiation marker expression in treated IVDs. (**a**–**c**) Intact discs were used as baseline and the difference in expression of anabolic (COL1, COL2 and ACAN) and catabolic (MMP3, MMP13 and ADAMTS4) genes of interest was analyzed in different regions of the IVD (NP, AFi, AFo). (**d**) When considering hMSC gene expression compared to the hydrogel loaded with 1 million hMSCs in the well-plate, KRT19 was significantly more expressed in IVDs treated with both 1 and 2 million hMSCs, thus demonstrating that the IVD microenvironment may promote hMSC differentiation towards the IVD phenotype. * *p* < 0.1, ** *p* < 0.05, *** *p* < 0.01, **** *p* < 0.001. NP = nucleus pulposus; IVD = intervertebral disc; hMSCs = human mesenchymal stem cells; AFi = inner annulus fibrosus; AFo = outer annulus fibrosus; COL1 = collagen type Iα1; COL 2 = collagen type IIα1; ACAN = aggrecan; MMP = matrix metalloproteinase; ADAMTS4 = a disintegrin and metalloproteinase with thrombospondin motifs 4; CD24 = cluster of differentiation 24; KRT19 = cytokeratin 19; CA12 = carbonic anhydrase 12.

In Figure 3, the effect of hMSC dose on the expression of anabolic (COL1, COL2 and ACAN) and catabolic (MMP3, MMP13 and ADAMTS4) markers in the IVD is reported. When looking at the overall gene expression pattern across the IVD tissues (Figure 3a–c), a trend towards ECM genes maintenance or up-regulation was noted in the NP and inner annulus fibrosus (AFi) and a down-regulation in the outer annulus fibrosus (AFo) compared to the intact IVD (baseline level in all graphs).

When looking at the NP tissue (Figure 3a), an increasing trend in collagen expression was observed in all groups (with or without hMSCs), which could indicate an attempt of rapid self-repair of the tissue following the nucleotomy.

Both in NP and AFi tissues (Figure 3a,b), the addition of the hydrogel without hMSCs induced a ~10× up-regulation of type 1 collagen and a ~10× down-regulation of aggrecan expression compared to the intact IVD. The addition of hMSCs led to a better preservation of the baseline gene expression of the intact IVD (represented by the line at 1 on all graphs), with lower COL1 and higher ACAN expression compared to the gel only group. Similar trends were observed with the two hMSC doses tested.

### 2.3. The Effects of Disc Microenvironment on Differentiation of hMSC within the HA/PRP/BTX Hydrogel

Consistent with previous ex vivo studies on hMSCs in IVD [20], the IVD microenvironment promoted hMSC differentiation towards the IVD phenotype by increasing a specific NP marker expression (Figure 3d). Results were calculated considering the hydrogel loaded with 1 million hMSCs and cultured in the well plate as a baseline. This effect seemed stronger when 2 million hMSCs were applied. Statistically significant changes were noted regarding KRT19 gene expression, which was significantly increased in IVDs treated with both 1 and 2 million hMSCs (*p* < 0.001) compared to the hydrogel cultured in the well plate.

## 3. Discussion

In this pilot study, we evaluated the effect of a HA/PRP/BTX hydrogel [22] as a carrier for hMSCs within the IVD environment using an ex vivo whole disc culture model [23]. Both HA and PRP have previously demonstrated promising results in the treatment of traumatic and degenerative musculoskeletal disorders and are now widely used in the clinical setting [24]. On the other hand, stem cell therapy for IDD is raising a great interest due to the increasing evidence of promoting tissue anabolism and—in some cases—regeneration in preclinical and clinical studies [25].

In a previous in vitro study, we have evaluated the biological and rheological properties of the HA/PRP/BTX hydrogel in combination with hMSCs. The hydrogel demonstrated to be easy to handle at room temperature and to rapidly polymerize at 37 °C, which would be ideal for its clinical application. After gelation, viscoelastic moduli progressively increased until reaching a plateau, acquiring an adequate biomechanical profile while not being harmful to cell activity and anabolism. The hydrogel construct promotes cell proliferation, glycosaminoglycan (GAG) synthesis, matrix gene expression and chondrogenic differentiation of hMSCs when cultured in presence of transforming growth factor-β1 (TGF-β1). Moreover, hMSCs within the scaffold demonstrated to be viable up to 21 days, thus confirming the cytocompatibility [18].

In the present study, we used a well-established bovine whole IVD organ culture model [20] to test the effect of the HA/PRP/BTX hydrogel encapsulating hMSCs in the disc environment. In the last decade, the use of bioreactors for whole IVD culture has become increasingly popular in intervertebral disc research due to several reasons. First, the setting of a 3D culture in a controlled and reproducible microenvironment mimicking the IVD niche has proved, unlike in vitro conditions, to maintain cell phenotype so as to better evaluate the interplay between IVD cells and matrix in physiological conditions. In addition, organ culture models are characterized by increased sustainability as they are in line with the 3R principle (Replacement, Reduction and Refinement) as well as cost-effective, thence reducing the burden of animal research [21]. Bioreactors have been extensively used to test innovative biomaterials, biomolecules and/or MSCs for NP regeneration. Indeed, ex vivo whole IVD models allow to easily deliver such therapeutic factors and to evaluate their effect under different conditions in term of mechanical loading and nutritional supply that can be opportunely manipulated based on the aim of the study [26]. In this way, the use of bioreactors may help fine-tune and refine animal models and boost the translation from bench to bedside.

In order to overcome the degenerative trigger caused by transannular injection of the hydrogel, we adopted a recently described strategy to access the NP directly from the endplate, hence avoiding the risk of leakage and/or extrusion of the construct [27]. This model has been previously tested for the ex vivo evaluation of different hydrogels for NP replacement and regeneration. Peroglio et al. evaluated a thermoreversible HA-poly(N-isopropylacrylamide) (HA-pNIPAM) hydrogel as an injectable carrier for hMSCs in vitro and ex vivo demonstrating a positive effect on ECM anabolism and differentiation towards a NP-like phenotype [3]. Using the same model, the role of biomechanical preconditioning on the response of IVD tissues to hMSCs embedded in either fibrin or saline solution was assessed. It was found that hMSCs promoted matrix anabolism and the expression of discogenic markers to a higher degree in IVDs cultured under degenerative conditions compared to IVDs cultured under physiological conditions [20]. Consistently with these investigations, our gel appeared to be integrated into the NP cavities with no signs of material extrusion.

We hypothesized that the HA/PRP/BTX hydrogel might provide hMSCs with a valid microenvironment promoting cell engraftment in the disc space, boosting anabolism and supporting differentiation towards a NP-like phenotype when injected into the IVD [18]. We assumed that the net effect of the hydrogel and cell construct would depend on both the IVD region (AFi, AFo, NP) and the cell dose delivered.

Our data indicate that hMSCs can survive inside the IVD (viability ~70%), although the viability was lower than in previous in vitro studies [20]. This could be explained by the absence of dynamic loading allowing metabolite transportation in physiological conditions. In addition, defect closure by cement sealing of the endplate might have also had a negative impact on metabolite exchange. Hence it is advisable to take these parameters in consideration in future studies. We observed a positive effect of hMSCs on the tissue adjacent to the hMSC-loaded gel construct (i.e., the region where the viability is mostly affected by the nucleotomy). Indeed, our present data indicate that the supply of a hMSC-loaded gel promoted cell viability in the adjacent tissue (independent of the dose, viability ~55% with hMSCs versus ~10% without hMSCs in the region 0–2 mm from the nucleotomy border), although further experiments are needed to confirm these trends. Furthermore, the fact that the disc cell viability further away from the gel (2–4 mm distance from the nucleotomy border) was better with a lower cell dose suggests that the addition of cells to a healthy, still well cellularized tissue may exceed the available metabolites. This could be different if hMSCs were applied into a degenerated human IVD, where the density of living cells is much lower than in a healthy juvenile bovine IVD. Hence, our data suggest that the ideal cell number to deliver is linked to the availability of nutrients inside the disc (which in turn is linked to the needs of IVD cells and to the tissue permeability).

We demonstrated the stimulating effect of the hMSCs on IVD cell viability, while gene expression did not greatly differ between IVDs injected with 1 or 2 million hMSCs, which may be a consequence of the close concentrations of hMSCs used. Safranin O staining demonstrated a stronger proteoglycan retention in the IVDs filled with hMSC-loaded hydrogels compared to hydrogel without hMSCs, suggesting a positive role of hMSCs in synthesizing or retaining sulphated proteoglycans inside the construct. Embedded hMSCs following intradiscal application showed an increased expression of a specific NP marker, namely KRT19, compared to embedded hMSCs cultured in vitro. According to previous investigations, KRT19 has been identified as a specific marker of notochordal ontogeny constantly expressed in human NP cells, thus supporting hMSC differentiation along the nucleopulpogenic lineage in this study. Conversely, no significant change of other NP markers (CA12 and CD24) was noted. CA12 is involved in maintaining the acid-base equilibrium in the highly acidic NP environment and has been reported to be specifically expressed by NP cells. However, CA12 expression seems to be affected by the degree of disc degeneration, with higher levels reported in the immature NP and lower levels in adult, non-degenerate NP [28]. Therefore, considering the absence of degenerative stimuli (e.g., excessive mechanical loading, pro-inflammatory culture media) and the short period between the nucleotomy and the collection of samples in our model, the lack of a significant alteration of CA12 expression may be reasonable. CD24 is a NP cell surface glycoprotein being highly expressed at the earliest stages of NP development. However, CD24 levels decrease with the acquisition of a mature NP phenotype and differential expression in human samples and following MSC differentiation has been reported in previous studies [29,30]. Collectively, these data confirm previous findings which suggest that the IVD environment can promote hMSC differentiation toward the NP phenotype [20]. Additionally, HA [31] and growth factors present in PRP [32] may further support this differentiation process.

The present study has some limitations. As IVDs were cultured in free swelling conditions without any biomechanical stimulation, further studies are needed to elucidate the role of mechanical loading with respect to hydrogel retention and integration as well as cell activity and metabolism. Indeed, it has been widely demonstrated that biomechanical stimuli in a physiological range promote hMSC chondrogenic differentiation and anabolism, while detrimental mechanical loading causes ECM breakdown and reduced cell viability [5]. Secondly, a broader spectrum of applied hMSC numbers could help to better appreciate the effect of this parameter on cell viability and differentiation of both the hMSC and surrounding IVD cells. Thirdly, part of the anabolic effect of the hydrogel may be associated with the release of growth factors contained in PRP. Therefore, the absence of a control group lacking PRP makes difficult to evaluate the isolated contribution of PRP itself to the net anabolic effect of the construct. Moreover, as the present study was preliminary and exploratory in nature, it was not possible to estimate about effect size and thus to build a structured power analysis. Therefore, sample size was calculated based on the law of diminishing return, as suggested for pilot investigations by previous studies [33]. Finally, the results obtained using the juvenile bovine tail model also need to be validated in a human, degenerated IVD.

## 4. Materials and Methods

### 4.1. Platelet-Rich Plasma

PLTs were obtained from whole blood drawn from healthy volunteer donors after being provided with informed consent. PRP was prepared according to the standard procedure used in clinical settings as previously described [18]. In terms of PLT count, PRP was ten times (10×) more concentrated than the whole blood (average 1.8 × 10^6^/μL). PLT count was determined with an automated hematologic system as suggested by pre-analytical recommendations. Total amount of PRP was subsequently aliquoted in 2 mL-volumes and frozen under controlled temperature (−20 °C). At the end of the procedure, sterility testing was performed on each batch of PRP. Collection, preparation and use of hemocomponents for non-transfusional use were performed using CE-marked devices for the specific purpose, according to the Directive 93/42/EC (class II or higher).

### 4.2. Hydrogel Assembly

HA 1.6%–32 mg/2 mL (molecular weight: 800–1200 kDa; Synovial Forte, IBSA, Lugano, Switzerland) was mixed with batroxobin (BTX; 5 BU/mL; Plateltex ACT, Plateltex S.R.O., Praha, Czech Republic) at 6:1 ratio. BTX is a gelling agent activated in presence of PRP and thus allows for constructing a three-dimensional hydrogel system encapsulating human hMSCs and growth factors contained in PRP. The HA/BTX blend was then mixed with PRP at a 1:1 ratio (final concentration volume ratio of the HA/PRP/BTX blend was 7:6:1) as previously described [34].

### 4.3. hMSCs Isolation and Culture

hMSCs were obtained from 2 bone marrow aspirates with written consent of the patients. hMSCs were isolated by Ficoll^®^ gradient centrifugation and adherence to tissue culture plastic from human vertebral bone marrow aspirates obtained with written consent from patients undergoing spine surgery. Cells were expanded under normoxic conditions (21% O_2_) in alpha-minimum essential medium (αMEM; Gibco-Invitrogen, Carlsbad, CA, USA) containing 100 U/mL penicillin, 100 µg/mL streptomycin, 10% fetal bovine serum (FBS; Pan Biotech, Aidenbach, Germany) and 5 ng/mL basic fibroblast growth factor (b-FGF; Peprotech, Rocky Hill, CT, USA). Early passage (P1–P2) hMSCs from two different donors (mean age: 43.5 ± 1.5 years old) were used in this study. Only bone marrow biopsies from vertebral bodies were included.

### 4.4. hMSC Encapsulation in the Hydrogel Scaffold

hMSCs were suspended in human PRP at the final concentration of either 10 × 10^6^/mL or 20 × 10^6^/mL. Then, 100 µL of PRP containing either 1 × 10^6^ or 2 × 10^6^ hMSCs were transferred to one vial containing 100 µL of the HA/BTX blend. The two components after gentle mixing were ready for injection.

### 4.5. Hydrogel Injection into IVDs and Organ Culture

**Figure 4:**
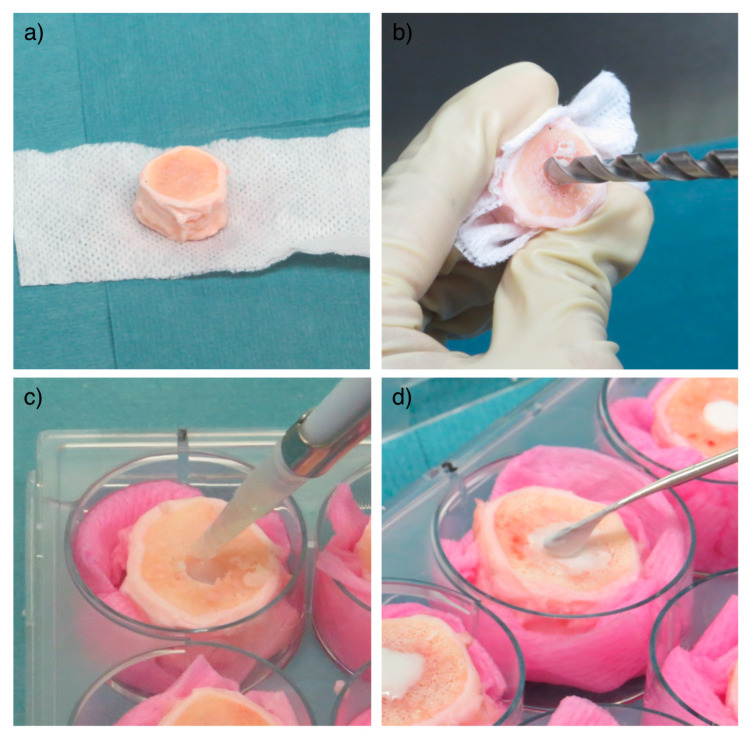
IVD preparation and injection with the HA/PRP/BTX hydrogel loaded with hMSCs. Soft tissues were carefully removed until obtaining a functional unit composed of the IVD together with surrounding endplates (**a**). Subsequently, a nucleotomy was performed through a 5 mm-defect created on the endplate surface using a drill (**b**). ~150 μL of the hydrogel were injected in the defect (**c**), which was then sealed with an endplate stopper and bone cement (**d**). HA = hyaluronic acid; PRP = platelet-rich plasma; BTX = batroxobin; hMSCs = human mesenchymal stem cells; IVD = intervertebral disc.

Bovine IVDs (*n* = 28) were obtained from 3 tails of 6–8-month-old calves purchased from a local abattoir as previously described [20]. Briefly, after soft tissue removal, IVDs comprising endplates were harvested using a bandsaw (Figure 4a). The endplates (1–2 mm thickness) were rinsed with Ringer solution using a Pulsavac jet-lavage system (Zimmer, Warsaw, Indiana, USA). The discs were further incubated in 1000 units/mL penicillin, 1000 µg/mL streptomycin in phosphate buffered saline (PBS) solution for 10 min. Subsequently, a nucleotomy was performed through a 5 mm-defect created on the endplate surface without violating the annulus fibrosus (AF) [27] (Figure 4b) and the cavity was filled with hMSCs suspended in ~200 µL of the PRP/HA/BTX scaffold (Figure 4c). Two doses of hMSCs were tested: 1 × 10^6^ and 2 × 10^6^ hMSCs per disc. The defect was then filled with an endplate stopper and sealed with bone cement (Figure 4d).

**Figure 5:**
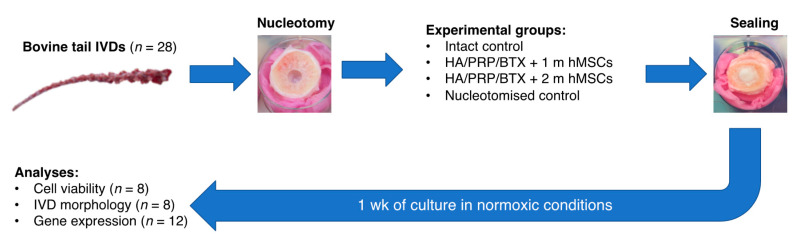
Schematic outline of the experimental design. IVD = intervertebral disc; HA = hyaluronic acid; PRP = platelet-rich plasma; BTX = batroxobin; hMSCs = human mesenchymal stem cells.

IVDs collected at the beginning of the experiment (day 0) were used as controls. Furthermore, nucleotomized IVDs filled with the hydrogel (without hMSCs) were included as additional control. Finally, hMSCs encapsulated in the hydrogel (1 and 2 × 10^6^ hMSCs per gel construct) were also cultured in well plates to evaluate the effect of the IVD environment on the hMSCs (Figure 5). IVDs were cultured in Dulbecco’s Modified Eagle Medium (DMEM; Life Technologies, Carlsbad, CA, USA) with 4.5 g/L glucose, 3.7 g/L sodium bicarbonate, 25 mM HEPES, 0.11 g/L sodium pyruvate, 1% penicillin/streptomycin, 2% fetal bovine serum (FBS; Life Technologies, Carlsbad, CA, USA), 1% insulin-transferrin-selenium (ITS; Sigma-Aldrich, Saint Louis, MO, USA), 0.2% Primocin (InvivoGen, San Diego, CA, USA). IVDs were cultured in free swelling conditions in six-well plates in the abovementioned medium for up to 7 days.

### 4.6. Histological and Cell Viability Analysis

Following one week of culture, the endplate opposite to the defect side was cut off with a scalpel and the IVDs were embedded in a cryocompound (Tissue-Tek©, O.C.T.TM, Sismex, Horgen, Switzerland) and snap-frozen. Transversal sections of each disc (*n* = 16 IVDs) were performed parallel to the endplate (10 µm-thick) using a microtome (HM 500 OMV, Microm, Walldorf, Germany). To get an overview over the whole IVD thickness, sections were collected in duplicates every 300 µm at 5 different locations (hence covering a height span of 1.5 mm). Samples were collected from two independent experiments.

Viability of the hMSCs and cells of the surrounding tissue was assessed by LDH/ethidium homodimer staining (*n* = 8). To assess cell viability, lactate dehydrogenase/ethidium homodimer staining was performed as previously described [23]. In brief, lactic acid, nicotinamide adenine dinucleotide and nitro blue tetrazolium chloride were added to a 40% Polypep solution (Sigma Aldrich, P5115) and adjusted to a pH of 8.0 using sodium hydroxide. Ethidium homodimer was diluted 1:1000 in PBS and added on 10 μm sections. After rinsing, the LDH-solution was added, and slides were left for 3 h at 37 °C before formalin fixation and mounting in aqueous-based medium.

Tiled images were acquired under brightfield and fluorescent signal in ten times magnification on a microscope (Olympus BX63, Nikkei, Japan). Image analysis was performed using ImageJ (version 2.1.0). Living and dead cells were counted in gel regions and in 0.5 mm² squares at 0-, 0.5-, 1-, 2-, 4- and 6-mm distances following a straight line from the cavity border. Thus, folded areas were avoided. Wherever possible, gel regions were analyzed centrally, and analysis of cell clusters was avoided due to the difficulty of distinction of single cells.

ECM was assessed by Safranin O/Fast Green staining (*n* = 8). Following fixation in 4% buffered formalin, Safranin O/Fast Green staining was performed to visualize proteoglycans (red) and collagen (green); cell nuclei were counterstained with hematoxylin (violet). Briefly, the slides were incubated with Weigert’s Hematoxylin for 30 min, then stained with 0.02% aqueous Fast Green (Fluka, Seelze, Germany) for 5 min and subsequently with 0.1% Safranin O (Kroma Gesellschaft, Munster, Germany) for 10 min. Sections were imaged as described above.

### 4.7. RNA Isolation and Gene Expression

RNA was extracted from tissues as previously described [35]. The following tissues were collected (*n* = 12): NP tissue in the intact control group (day 0), the remaining NP in the nucleotomized control group with hydrogel, the remaining NP plus the hydrogel containing hMSCs in the experimental groups (1 × 10^6^ and 2 × 10^6^ hMSCs). For all groups, AFi and AFo were also collected from each IVD. A piece of each tissue (≈ 150 mg) was placed into a 15 mL tube after being minced in small fragments with a scalpel. Samples were hydrated with phosphate buffered saline (PBS; 5 mL/tube) and centrifuged at 500 g for 2 min. After PBS removal, tissues were digested in DMEM containing 2 mg/mL pronase (Roche, Mannheim, Germany) at 37 °C for 1 h on a waving shaker (30 rpm; Polymax 1040, Heidolph, Schwabach, Germany). Samples were centrifuged at 300 rpm for 2 min and the digestion process was then arrested by adding 0.5 mL FBS/5 mL solution; tissues were washed twice with PBS. Subsequently, after PBS aspiration, samples were snap-frozen with liquid nitrogen for 30 s and pulverized with a pestle. Powdered fragments were then moved to 2 mL tubes containing 1 mL TRI reagent and 5 µL polyacryl carrier (Qiagen, Hilden, Germany) and frozen at -80 °C until total RNA extraction. The same procedure was applied to extract RNA from hMSCs in hydrogels cultured in the IVD cavity and in the well plates.

cDNA synthesis was performed with SuperScript Vilo Synthesis kit (Invitrogen, ThermoFisher Scientific, Carlsbad, CA, USA), and real-time PCR was performed utilizing TaqMan Gene Expression Master Mix (Applied Biosystems, Foster City, CA, USA). Data were acquired with QuantStudio 6 Flex instrument (Applied Biosystems) and analyzed using the ddCt method. The following genes were analyzed (Table 1): collagen type Iα1 (COL1), collagen type IIα1 (COL2), aggrecan (ACAN), matrix metalloproteinase 3 (MMP3) and 13 (MMP13), a disintegrin and metalloproteinase with thrombospondin motifs 4 (ADAMTS4), cytokeratin 19 (KRT19), carbonic anhydrase 12 (CA12) and cluster of differentiation 24 (CD24). The 18S ribosomal RNA (18S) and glyceraldehyde 3-phosphate dehydrogenase (GAPDH) were used as endogenous controls. For the assessment of IVD tissues (NP, inner AF and outer AF), tissues collected from the IVDs cultured for the same period of time were used as reference. For the assessment of hMSCs in hydrogel, the 1 million hMSC seeded hydrogel cultured in the well plate for one week was used as reference sample.

### 4.8. Statistical Analysis

Statistical analysis was performed using Graph Pad Prism version 7.0 (GraphPad Software; San Diego, CA, USA). After confirming normal distribution of the data, Two-way ANOVA with Tukey post-hoc for multiple-group comparisons and Student’s t test for two-group comparisons were performed (*p* < 0.1 trend, *p* < 0.05 significant difference). Regarding gene expression, 2-ddCt data were log2 transformed prior to statistical analysis. Number of samples for each experimental group was calculated on the basis of the “Resource Equation”: N = (E × T) − T; (10 ≤ E ≤ 20), where E is the number of samples for each group and T is the number of the experimental groups. In our study, sample size calculated using this method was 24, which is considered adequate to obtain significant results according to our study design [33].

## 5. Conclusions

This study demonstrated that hMSCs embedded in a hydrogel composed of HA/PRP/BTX can promote cell engraftment and allows hMSC differentiation toward a NP-like phenotype supported by the disc microenvironment. Furthermore, after 1 week of culture, hydrogels showed signs of integration with surrounding tissues. As HA and PRP are routinely employed in the clinical setting, this construct holds a significant translational potential. Further ex vivo studies involving biomechanical stimulation of transplanted IVDs and subsequent in vivo investigations are needed to provide further evidence for IVD regeneration using this innovative hydrogel.

## Figures and Tables

**Table 1:** List of genes analyzed by RT-PCR.

COL1	Primer fw (5′-3′)	CCC TGG AAA GAA TGG AGA TGA T
	Primer rev (5′-3′)	ACT GAA ACC TCT GTG TCC CTT CA
	Probe (5′ FAM/3′TAMRA)	CGG GCA ATC CTC GAG CAC CCT
COL2	Primer fw (5′-3′)	GGC AAT AGC AGG TTC ACG TAC A
	Primer rev (5′-3′)	GAT AAC AGT CTT GCC CCA CTT ACC
	Probe (5′ FAM/3′TAMRA)	CCT GAA GGA TGG CTG CAC GAA ACA TAC
ACAN	Primer fw (5′-3′)	AGT CCT CAA GCC TCC TGT ACT CA
	Primer rev (5′-3′)	CGG GAA GTG GCG GTA ACA
	Probe (5′ FAM/3′TAMRA)	CCG GAA TGG AAA CGT GAA TCA GAA TCA ACT
MMP3	Gene expression assay	Hs00968305_m1 (Applied Biosystems)
MMP13	Primer fw (5′-3′)	CGG CCA CTC CTT AGG TCT TG
	Primer rev (5′-3′)	TTT TGC CGG TGT AGG TGT AGA TAG
	Probe (5′ FAM/3′TAMRA)	CTC CAA GGA CCC TGG AGC ACT CAT GT
ADAMTS4	Gene expression assay	Hs00192708_m1 (Applied Biosystems)
KRT19	Gene expression assay	Hs_00761767_s1 (Applied Biosystems)
CA12	Gene expression assay	Hs00154221_m1 (Applied Biosystems)
CD24	Gene expression assay	Hs00273561_s1 (Applied Biosystems)
18S rRNA	Assay reagents	4310893E (Applied Biosystems)
GAPDH	Gene expression assay	Hs02786624_g1 (Applied Biosystems)

RT-PCR = real-time polymerase chain reaction; COL1 = collagen type Iα1; COL2 = collagen type IIα1; ACAN = aggrecan, MMP3 = matrix metalloproteinase 3; MMP13 = matrix metalloproteinase 13; ADAMTS4 = a disintegrin and metalloproteinase with thrombospondin motifs 4; KRT19 = cytokeratin 19; CA12 = carbonic anhydrase 12; CD24 = cluster of differentiation 24; GAPDH = glyceraldehyde 3-phosphate dehydrogenase.

## Abbreviations

ACAN	Aggrecan
ADAMTS	A disintegrin and metalloproteinase with thrombospondin motifs
AF	Annulus fibrosus
AFi	Inner annulus fibrosus
AFo	Outer annulus fibrosus
BTX	Batroxobin
CA	Carbonic anhydrase
CD	Cluster of differentiation
COL1	Collagen type Iα1
COL2	Collagen type IIα1
ECM	Extracellular matrix
GAG	Glycosaminoglycan
GAPDH	Glyceraldehyde 3-phosphate dehydrogenase
HA	Hyaluronic acid
HA-pNIPAM	HA-poly(N-isopropylacrylamide)
hMSCs	Human mesenchymal stem cells
IDD	Intervertebral disc degeneration
IVD	Intervertebral disc
KRT	Cytokeratin
LDH	Lactate dehydrogenase
LBP	Low back pain
MMP	Matrix metalloproteinase
NP	Nucleus pulposus
NF-κB	Nuclear factor kappa-light-chain-enhancer of activated B cells
OA	Osteoarthritis
PLTs	Platelets
PRP	Platelet-rich plasma
RT-PCR	Real-time polymerase chain reaction
TGF-β1	Transforming growth factor-β1
